# Vernolactone Promotes Apoptosis and Autophagy in Human Teratocarcinomal (NTERA-2) Cancer Stem-Like Cells

**DOI:** 10.1155/2019/6907893

**Published:** 2019-12-19

**Authors:** Nuwanka K. Abeysinghe, Ira Thabrew, Sameera R. Samarakoon, Meran K. Ediriweera, Kamani H. Tennekoon, Varuni P. C. Pathiranage, Anuka S. Mendis

**Affiliations:** Institute of Biochemistry, Molecular Biology and Biotechnology (IBMBB), University of Colombo, No. 90, Cumaratunga Munidasa Mawatha, Colombo 03, Sri Lanka

## Abstract

*Vernonia zeylanica*, is a shrub endemic to Sri Lanka. *V. zeylanica* has been used in Sri Lankan traditional medicine for the treatment of various diseases and conditions. The present study was designed to determine antiproliferative, apoptotic, autophagic, and antioxidant effects of vernolactone, isolated from *V. zeylanica*, in human embryonal carcinoma cells (NTERA-2, a cancer stem cell model). Antiproliferative effects of vernolactone in NTERA-2 cells and human peripheral blood mononuclear cells (control cells) were evaluated using the Sulforhodamine B (SRB) assay and WST-1 antiproliferative assays, respectively. The antiproliferative effect of vernolactone was further investigated using the colony formation assay. Effects of vernolactone on apoptosis were investigated by phase contrast light microscopic and fluorescence microscopic analysis, caspase 3/7 expression, and real-time PCR of apoptosis-associated genes *p53* and *Survivin*. The effect of vernolactone on NTERA-2 cell migration was monitored using the wound healing assay. Effects of vernolactone on the expression of autophagy-related genes (*LC3*, *Beclin 1*, *PI3K*, *Akt*, and *mTOR*) were evaluated using real-time PCR. 2,2-Diphenyl-1-2,2-diphenyl-picrylhydrazyl (DPPH) radical scavenging assay, 2,2′-azinobis-(3-ethylbenzothiazoline-6-sulfonic acid) (ABTS) radical scavenging, and ferric reducing antioxidant power (FRAP) assays were also carried out to evaluate the antioxidant activity of vernolactone. Overall results confirm that vernolactone can exert antiproliferative effects, induce apoptosis and autophagy, and decrease NTERA-2 cell migration in a dose- and time-dependent manner with a very small antioxidant property.

## 1. Introduction

Despite the advances in cancer therapeutics, millions of people around the world are diagnosed with various types of cancer each year and half of the patients fail to survive [1]. A number of genetic mutations and several epigenetic alterations have been identified as major factors responsible for the development and progression of cancer [2]. Indefinite proliferation, aberrant growth factor signaling pathways, and resistance to chemo- and radiotherapies make it hard to find a permanent cure for cancer [3]. It is evident that cancer stem cells (CSCs), a unique subpopulation of cancer cells, are involved in the initiation and maintenance of primary tumors [4]. Recent discoveries have identified CSCs as the major cause of chemotherapy resistance, thereby allowing tumor relapses and metastasis [5]. Irregularities in signaling pathways such as Wnt/*β*-catenin, Notch, and Hedgehog are common in CSCs, and these irregularities in signaling pathways provide a strong rationale to investigate new cancer stem cell therapeutics [6].

Naturally derived anticancer drugs are widely employed in the development of anticancer treatments as these drugs exhibit higher specificity and lesser side effects compared to synthetic drugs [7–9]. Natural drugs have been reported to demonstrate antitumor activities via distinct mechanisms such as modulation of survival signaling pathways, induction of apoptosis and autophagy, inhibition of angiogenesis, and removal of oxidative stress [10]. *Vernonia zeylanica* is a plant endemic to Sri Lanka which has been used in the traditional Sri Lankan medicine to treat several diseases and conditions including boils, asthma, bone fractures, and food poisoning [11]. A recent study conducted in our laboratory demonstrated that combined chloroform and ethyl acetate extracts of *V. zeylanica* can induce antiproliferative effects in three breast cancer cell lines. This work leads to the isolation of vernolactone, a new sesquiterpene lactone, from *V. zeylanica* that has shown cytotoxic and apoptotic effects in three different breast cancer phenotypes through modulating heat shock proteins [12].

The present study was designed to investigate the potential apoptotic and autophagic effects of vernolactone in NTERA-2 cl.D1 teratocarcinomal (NTERA-2) cancer stem-like cells. Embryonal carcinoma cells, which are derived from teratocarcinomas (most commonly occurring in the testis), are considered the malignant counterparts of pluripotent embryonic stem cells [13]. Therefore, the undifferentiated, pluripotent embryonal carcinoma cells have been reported as the most convenient tool to investigate the fundamental molecular mechanisms of embryonic stem cells *in vitro* [14, 15]. NTERA-2 cl.D1 is a completely characterized highly pluripotent cancer stem cell line that has a close resemblance to human embryonic stem cells [16].

## 2. Methodology

### 2.1. General

Moloney murine leukemia virus (M-MLV) reverse transcriptase was purchased from the Promega Corporation, Madison, USA. Powdered Dulbecco's modified Eagle medium, fetal bovine serum (FBS), streptomycin/penicillin, dimethyl sulfoxide (DMSO), agarose and trypsin/EDTA, DPPH (2,2-diphenyl-1-picryl hydrazyl), paclitaxel, ABTS (2,2-azino-bis(3-ethylbenzothiazoline-6-sulfonic acid) diammonium salt), aluminum chloride, trolox (6-hydroxy-2-5-7-8-tetramethylchroman-2-carboxylic acid), and Histopaque®-1077 were purchased from the Sigma-Aldrich Chemical Company, St. Louis, MO, USA. A TRIzol reagent was purchased from the Invitrogen Life Technologies, Carlsbad, CA, USA. All-trans retinoic acid (ATRA) was purchased from Alfa Aesar, Lancashire, U.K. Human embryonal carcinoma (NTERA-2) cells were purchased from the American Type Culture Collection (ATCC), Manassas, VA, USA. PCR primers were purchased from the Integrated DNA Technologies (IDT), Coralville, IA, USA.

### 2.2. Cell Culture

NTERA-2 cells were cultured in Dulbecco's modified Eagle medium (DMEM) supplemented with 10% fetal bovine serum (FBS), 50 IU/mL penicillin, and 50 *μ*g/mL streptomycin antibiotic mixtures according to the ATCC recommendations. Cells were maintained at 37°C in 95% air and 5% CO_2_ atmosphere with 95% humidity.

### 2.3. Sulforhodamine B (SRB) Assay

The Sulforhodamine B (SRB) assay was carried out according to the reported method by Vichai and Kirtikara [17] with modifications described by Samarakoon et al. [18]. NTERA-2 cells were grown in T_75_ flasks prior to the assay. Cells were then trypsinized, seeded in 96-well plates (5 × 10^3^ cells per well) containing DMEM, and incubated for 24 h. Following incubation, cells were treated with different concentrations of vernolactone (3.125–50 *μ*g/mL) and paclitaxel (0.0625–10 *μ*g/mL) and incubated for 24, 48, and 72 h. After the incubation period, cells were fixed with 40 *μ*L of ice-cold 50% trichloroacetic acid (TCA). Plates were incubated for 1 h at 4°C and washed five times with tap water. Fixed cells were air dried and stained with 50 *μ*L of 0.4% (*w*/*v*) SRB dye for 15 min at room temperature. After incubation, the unbound dye was removed by washing the cells five times with 1% acetic acid. The protein-bound SRB dye was solubilized by adding unbuffered tris base (100 *μ*L) to each well and placing the plates on a plate shaker for 1 h at room temperature. The absorbance values were recorded at 540 nm using a microplate reader (Synergy HT microplate reader, BioTek Instruments, USA). The percentage of cell viability was calculated as follows: percentage of cell viability = {(At‐Ab)/(Ac‐Ab)} × 100, where At is the absorbance value of the treated sample, Ab is the absorbance value of the blank, and Ac is the absorbance value of the control (untreated) sample. Finally, the half maximum inhibitory concentration (IC50) was calculated using the GraphPad Prism 7.00 (GraphPad Software Inc., San Diego, CA, USA).

### 2.4. Isolation of Peripheral Blood Mononuclear Cells (PBMC)

Peripheral blood mononuclear cells (PBMC) were used as the normal control cells to evaluate the antiproliferative effect of vernolactone and paclitaxel. PBMC were isolated according to the method described by Tharmarajah et al. [19] with slight modifications. Collected venous blood (3 mL) was carefully layered onto an equal amount of 3 mL of Histopaque-1077 and centrifuged at 400 × *g* for 30 min at room temperature. Following centrifugation, the opaque interface containing mononuclear cells were carefully transferred to a clean conical centrifuge tube and cells were washed with isotonic phosphate-buffered saline (PBS) (10 mL). Cells were then resuspended in RPMI 1640 cell culture medium and seeded into 96-well plates (5 × 10^4^ cells per well) and incubated for 24 h. After incubation, cells were treated with different concentrations of vernolactone (3.125–50 *μ*g/mL) and paclitaxel (0.0625–10 *μ*g/mL) and incubated for 24, 48, and 72 h. The WST-1 cell proliferation assay was performed according to the manufacturer's instructions to evaluate antiproliferative effects of vernolactone and paclitaxel in PBMC.

### 2.5. Colony Formation Assay

The colony formation assay was performed according to the previously reported method by Frankan et al. [20] with some modifications. NTERA-2 cells (500 cells/mL) were seeded in 96-well plates and incubated for 24 h. After incubation, cells were treated with different concentrations of vernolactone (3.125–50 *μ*g/mL) and paclitaxel (0.0625–10 *μ*g/mL) and incubated for 7 days. Following 7 days of incubation, cell colonies were counted after staining with the SRB dye.

### 2.6. Cell Migration Assay (Wound Healing Assay)

The effect of vernolactone and paclitaxel on NTERA-2 cell migration was determined using the cell migration assay [21]. NTERA-2 cells (2 × 10^5^ cells/mL) were seeded in 24-well plates and incubated until confluence. In a sterile environment, a vertical wound was made through the cell monolayer using a sterile pipette tip. After making a wound through the cell monolayer, the medium was aspirated and wells were washed with culture medium to remove cell debris. Different concentrations of vernolactone (1, 2, and 4 *μ*g/mL) and paclitaxel (0.25, 0.5, and 1 *μ*g/mL) were added to each well and incubated to avoid detaching additional cells. Initial pictures of each well, just after the treatment, were taken from an inverted phase contrast light microscope. Then the plates were incubated at 37°C in 95% air and 5% CO_2_ atmosphere with 95% humidity. At several time points, the plates were removed from the incubator and snap shot pictures were taken to check for wound closure. The width of the wound was measured using a scale bar to analyze the rate of migration.

### 2.7. DPPH Assay

The method used by Chan et al. [22] was used for the DPPH assay with slight modifications. A dilution series of vernolactone (0.1220 *μ*g/mL–1000 *μ*g/mL) was prepared prior to the assay. DPPH (60 *μ*L, 2 mg/mL) and methanol (90 *μ*L) were added to each well containing 50 *μ*L of vernolactone, and plates were incubated in the dark for 10 min. Trolox was used as the positive control. Following incubation, absorbance of each well was recorded at 517 nm using a microplate reader. Percentage free radical scavenging activity was calculated using the following formula: percentage of free radical scavenging ability = (Abcontrol–Absample)/(Abcontrol) × 100.

### 2.8. ABTS Radical Scavenging Assay

ABTS radical scavenging ability of vernolactone was measured using the ABTS radical scavenging assay [23]. A dilution series of vernolactone (7.8125 *μ*g/mL–1000 *μ*g/mL) in PBS was prepared before the assay. ABTS (40 *μ*L) and vernolactone (160 *μ*L) at various concentrations were added to each well, and plates were incubated at room temperature for 10 min. Trolox was used as the positive control. Following incubation, absorbance of each well was recorded at 734 nm using a microplate reader and percentage radical scavenging activity was calculated using the following formula: percentage of free radical scavenging ability = (Abcontrol–Absample)/(Abcontrol) × 100.

### 2.9. Ferric Reducing Antioxidant Power (FRAP) Assay

Prior to the assay, a FRAP reagent [300 mM acetate buffer (pH 3.6), 20 mM ferric chloride, and 10 mM 2,4,6-tripyridyl-s-triazine (TPTZ) in a ratio of 10 : 1 : 1] and a dilution series of vernolactone (7.8125 *μ*g/mL–1000 *μ*g/mL) in acetate buffer were prepared. Vernolactone (20 *μ*L from each concentration), FRAP reagent (150 *μ*L), and acetate buffer (30 *μ*L) were mixed together and incubated at room temperature for 8 min. Following incubation, absorbance of each well was recorded at 600 nm using a microplate reader. FRAP assay was conducted according to the previously described protocol by Benzie and Strain [24] with slight modifications. Results were expressed as mg trolox equivalent/g of extract.

### 2.10. Fluorescent Microscopic Analysis

NTERA-2 cells (2 × 10^5^ cells/mL) were cultured on cell culture-treated cover slips and incubated for 24 h. Cells were then treated with different concentrations of vernolactone (2, 4, and 8 *μ*g/mL) and paclitaxel and further incubated for 24 h. After incubation, cells were fixed with 4% formaldehyde (1 mL) and stained with acridine orange (AO)/ethidium bromide (EB) and Hoechst 33258 dye as described by Ediriweera et al. [25]. Cell images were captured using a fluorescence microscope (Olympus BX 51 TRF, Japan).

### 2.11. Caspase Glo® 3/7 Assay

NTERA-2 cells (2 × 10^4^ cells per well) were seeded in 96-well plates and incubated for 24 h. Prior to the caspase assay, cells were treated with different concentrations of vernolactone (0.5, 1, 2, and 4 *μ*g/mL) and paclitaxel (0.25, 0.5, 1, and 2 *μ*g/mL) and incubated for 24 h. Following incubation, Caspase Glo® 3/7 was conducted according to the manufacturer's instructions. Percentage of caspase activity was determined according to the following formula: percentage of caspase activity = {(Lt‐Lb)/(Lc‐Lb)} × 100, where Lt is the luminescence value of treated sample, Lb is the luminescence value of blank, and Lc is the luminescence value of control.

### 2.12. Gene Expression Analysis

NTERA-2 cells (2.5 × 10^5^ cells/mL) were cultured in T_25_ cell culture flasks and incubated for 24 h. Cells were then treated with different concentrations of vernolactone (2 and 4 *μ*g/mL) and paclitaxel (2 and 4 *μ*g/mL) and incubated for 24 h. RNA was extracted using the TRIzol® reagent. The extracted RNA was used to synthesize complementary DNA (c-DNA). Real-time PCR reactions were carried out in Stratagene Mx3000P. Each real-time PCR reaction contained 12.5 *μ*L of MESA GREEN qPCR Master Mix, 0.5 *μ*L of forward and reverse primers, 2 *μ*L of c DNA, and 9.5 *μ*L of PCR water. Real-time PCR cyclic conditions for *GAPDH*, *p53*, and *Survivin* were adapted from the study reported by Ediriweera et al. [26]. Primers *LC3*, *Beclin 1*, *PI3K*, *Akt*, and *mTOR* were optimized before the real-time PCR experiments. Real-time PCR cycling conditions for genes *GAPDH*, *p53*, *Survivin*, and *LC3* were as follows: initial denaturation for 10 min at 94°C and amplification in three steps for 35 cycles (denaturation for 30 sec at 94°C, annealing for 1 min at 58°C, and extension for 1 min at 72°C). Annealing temperature for the genes *Beclin 1*, *PI3K*, *Akt*, and *mTOR* was maintained at 52°C. The method developed by Livak and Schmittgen was used to analyze the results of real-time PCR [27].

### 2.13. Cellular Differentiation

For cell differentiation, cell differentiation analysis via cell aggregation described by Paquet-Durand et al. [28] was performed with some modifications. All-trans retinoic acid (ATRA) was used as the positive control to induce the cellular differentiation. NTERA-2 cells (5 × 10^5^ cells/mL) were seeded in nontreated 24-well plates. After overnight incubation, cell aggregates were treated with 2 and 4 *μ*g/mL of vernolactone and 10 *μ*M (3.0044 *μ*g/mL) of ATRA, as it was previously reported [29], to give a high yield of postmitotic neurons. The medium was replaced, and cells were replated in new wells to induce neurite formation. The medium with the above drug concentrations was replaced every 2-3 days by transferring the cell suspension into centrifuge tubes and centrifuging at 150 × *g* for 7 min. Cells were incubated for 10 days to observe the neurite formation.

### 2.14. Statistical Analysis

All the experiments were carried out in triplicates, and Graph Pad Prism 7.00 software (GraphPad Software Inc., San Diego, CA, USA) was used to perform the statistical analysis. The significant difference in caspase assay was determined by the one way analysis of variance (ANOVA) with Bonferroni's posttest while the significant differences in gene expression analysis were determined by one way analysis of variance (ANOVA) with Dunnett's posttest and the differences were considered to be statistically significant at *P* < 0.05.

## 3. Results and Discussion

### 3.1. Antiproliferative Effects of Vernolactone

#### 3.1.1. Antiproliferative Effects of Vernolactone in NTERA-2 and Peripheral Blood Mononuclear Cells

As evident from the results of the SRB assay, vernolactone can exert a potential dose- and time-dependent inhibition of NTERA-2 cell proliferation similar to the positive control paclitaxel. However, vernolactone and paclitaxel had demonstrated relatively less antiproliferative effects in peripheral blood mononuclear cells (PBMC), the noncancerous control cells of NTERA-2 cells, as indicated by the WST-1 cell proliferation assay (Table 1).

#### 3.1.2. Colony Formation Assay

**Figure 1:**
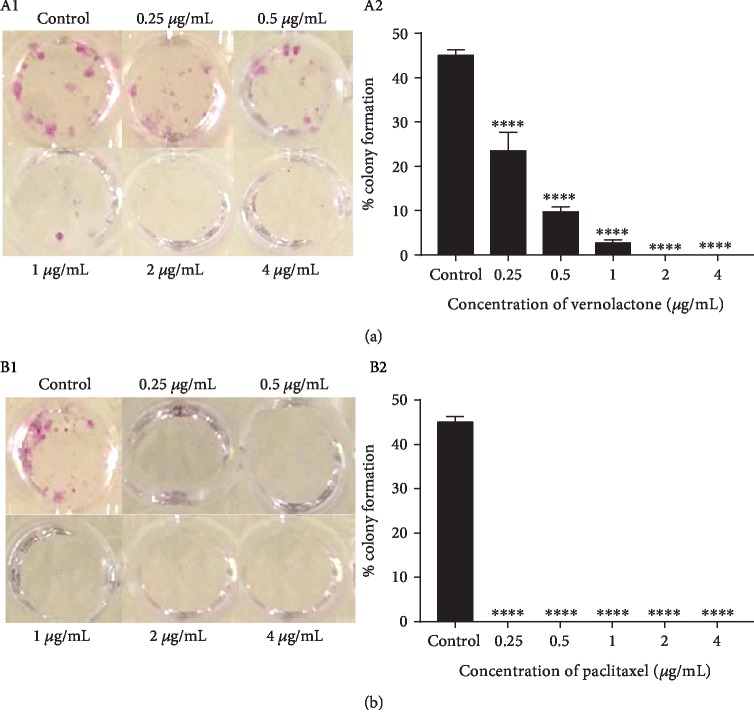
(a) (A1) Appearance of wells in a 96-well plate containing NTERA-2 cells fixed with TCA and stained with the SRB dye, following seven days of vernolactone treatment. (A2) Average colony formation rates of vernolactone-treated NTERA-2 cells. (b) (B1) Appearance of wells in a 96-well plate containing NTERA-2 cells fixed with TCA and stained with SRB dye, following seven days of paclitaxel treatment. (B2) Average colony formation rates of paclitaxel treated NTERA-2 cells. ^∗∗∗∗^*P* < 0.0001.

The antiproliferative effect of vernolactone was further supported by the results of the colony assay. This assay enables to determine the differences in reproductive viability between the untreated control cells and cells that have been exposed to a testing drug [30, 31]. After seven days of drug treatment, vernolactone-treated NTERA-2 cells displayed a dose-dependent reduction in the colony formation rates when compared to the untreated control. The cells were observed under a phase contrast light microscope upon staining with the SRB dye (Figure 1(a)). In contrast, paclitaxel- (positive control) treated NTERA-2 cells displayed 0% of colony formation rates at all the concentrations tested, upon staining with the SRB dye (Figure 1(b)). Single cells which have lost their ability to divide and proliferate could be observed under a phase contrast light microscope. Those paclitaxel-treated cells which were not visible to the naked eye have undergone reproductive death. Thus, these results showed that vernolactone can strongly inhibit the colony formation ability of NTERA-2 cells in a dose- and time-dependent manner when compared to the positive control.

#### 3.1.3. Analysis of Migration Rate of NTERA-2 Cells

**Figure 2:**
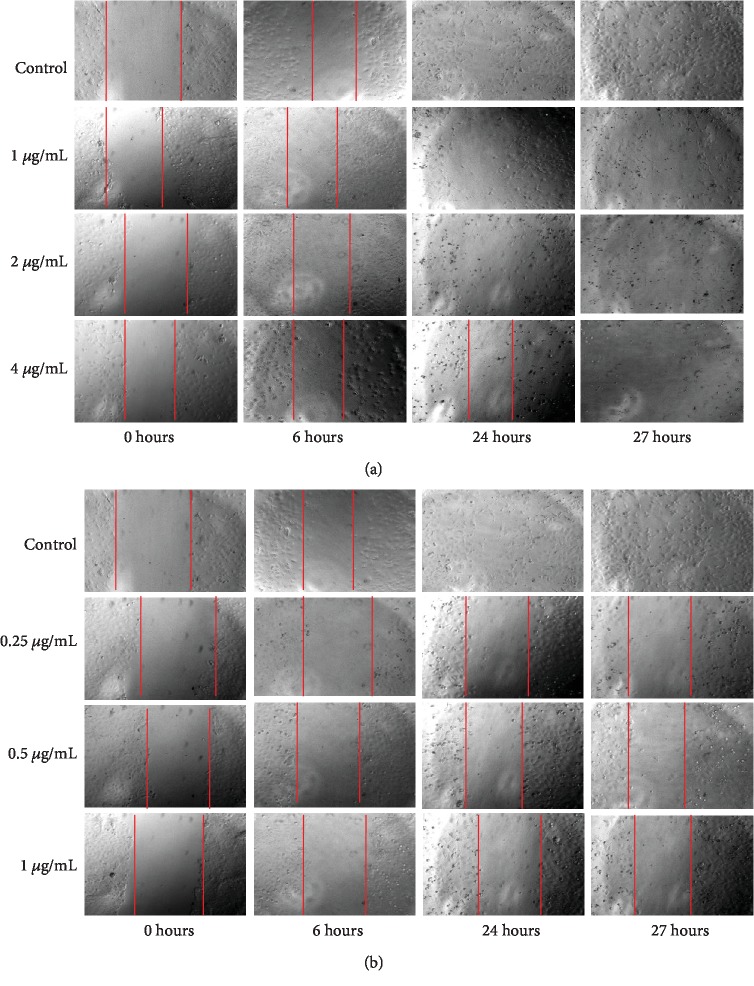
Phase contrast light microscopic images captured at different time points after creating the gap, followed by (a) vernolactone and (b) paclitaxel treatments.

**Figure 3:**
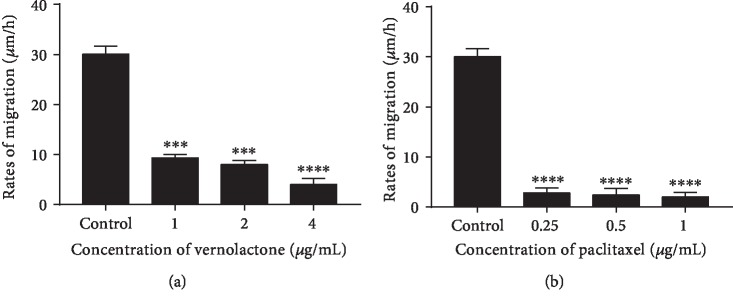
Cell migration rates of (a) vernolactone- and (b) paclitaxel-treated NTERA-2 cells. ^∗∗∗^*P* < 0.001 and ^∗∗∗∗^*P* < 0.0001.

Cell migration is an important measurement in cancer research that reveals informative details about the migratory behaviors of cancer cells. Cell migration wound healing assay determines the ability of a cell line to migrate and close the gap created artificially in a confluent monolayer of cells [21, 32]. Migration rates were observed to be dose dependently decreased in the NTERA-2 cells treated with vernolactone when compared to the untreated control cells (Figures 2(a) and 3(a)). Although some of the cells have migrated in vernolactone-treated samples, those cells have lost their viability as the incubation time increased. Paclitaxel-treated cells have also demonstrated a very low cell migration rate (less than 3 *μ*m/h for all concentrations), where the created gap had almost the same distance as at the beginning (Figures 2(b) and 3(b)).

### 3.2. Antioxidant Effects of Vernolactone

Free radicals are highly reactive species capable of damaging biologically important cellular molecules and play a major role in cancer development. Antioxidants are a group of chemicals that neutralize free radicals by interacting with them, thus preventing the damage caused by them. Several assays have been used to determine the antioxidant capacities of pure compounds, plant extracts, and food materials. This study has used three different methods: DPPH radical scavenging activity, ABTS radical-scavenging activity, and ferric reducing antioxidant power (FRAP) assay to evaluate the antioxidant activity of vernolactone [33–36]. Vernolactone demonstrated a very low free radical scavenging activity (IC_50_ > 1000 *μ*g/mL) in both the DPPH and ABTS assays when compared to the positive control (Trolox) that showed a higher free radical scavenging activity (IC_50_ = 2.8494 *μ*g/mL and 4.29 *μ*g/mL, respectively). When FRAP assay was performed with vernolactone, it also appeared to lack the total reducing power to change the absorbance of the reaction mixture confirming that vernolactone had low antioxidant potential. According to the results obtained from DPPH, ABTS, and FRAP assays, it is evident that antioxidant activity is not a mechanism by which vernolactone exerts antiproliferative activity in NTERA-2 cells.

### 3.3. Effects of Vernolactone on Apoptosis

Apoptosis, a mechanism of programmed cell death, is a key regulator of development of cellular homeostasis [37]. A loss of balance between cell division and cell death occurs in cancer is one of the essential hallmarks in a cell that cause malignant transformation [38]. A series of characteristic morphological changes occur in cancer cells undergoing apoptosis, due to consequences of several molecular and biochemical events. These include cell rounding, plasma membrane blebbing, reduction in cell volume, nuclear chromatin condensation, DNA fragmentation, and large cells which break up into apoptotic bodies, which lead to marked cell shrinkage [39].

#### 3.3.1. Fluorescent Microscopic Observations

**Figure 4:**
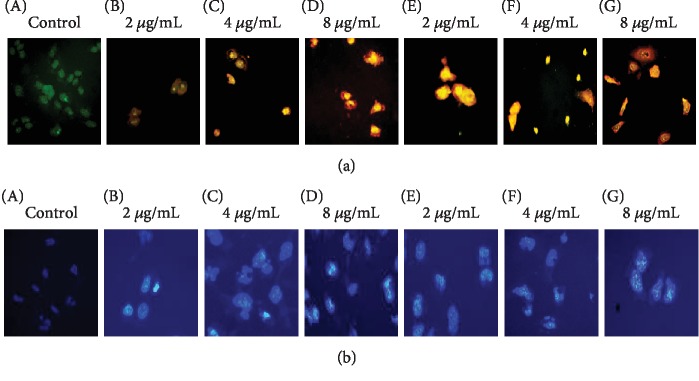
Fluorescent microscopic observations of NTERA-2 cells exposed to vernolactone and paclitaxel for 24 h. (a) Acridine orange (AO) and ethidium bromide (EB) and (b) Hoechst 33258 dye (using a blue filter) (magnification 200x): A—untreated control (0.1% DMSO); B—vernolactone (2 *μ*g/mL); C—vernolactone (4 *μ*g/mL); D—vernolactone (8 *μ*g/mL); E—paclitaxel (2 *μ*g/mL); F—paclitaxel (4 *μ*g/mL); G—paclitaxel (8 *μ*g/mL).

Supports for the observed morphological changes for vernolactone with phase contrast light microscopy were further confirmed by fluorescent microscopic observations with different fluorescent DNA binding dyes (AO/EB and Hoechst 33258 dyes). AO/EB staining gives a clear identification of apoptosis-associated changes of cell membranes during apoptosis and accurately distinguishes the different stages of apoptosis by staining early apoptotic cells in yellow and late apoptotic cells in red colour depending on the degree of loss of the cell membrane. An increase in induction of apoptosis was observed in both vernolactone- and paclitaxel-treated NTERA-2 cells (2, 4, and 8 *μ*g/mL) in a dose-dependent manner (Figure 4(a)).

Nuclear damages occur in apoptotic cells were detected by Hoechst 33258 staining with light blue colour-condensed nuclei. A dose-dependent increase in condensed and fragmented DNA was clearly visible in both vernolactone- and paclitaxel-treated NTERA-2 cells when compared to intact nuclear structures of untreated control cells (Figure 4(b)).

#### 3.3.2. Expression of Caspase 3 and Caspase 7 in NTERA-2 Cells

**Figure 5:**
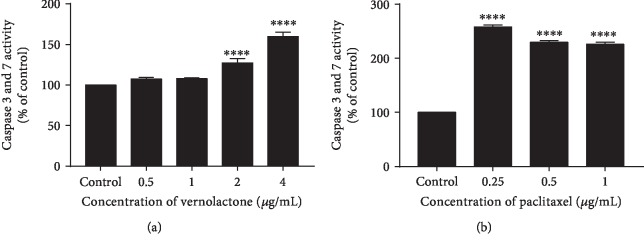
Expression of caspase 3 and caspase 7 in NTERA-2 cells after 24 hours of vernolactone (a) and paclitaxel (b) treatments. ^∗∗∗∗^*P* < 0.0001.

The Caspase Glo® 3/7 assay is a homogeneous, luminescent assay which measures the activity of caspase 3 and caspase 7. These members belong to the caspase family, a cysteine-dependent aspartate-specific protease family that mediates proteolysis and specifically activates apoptotic cells. Vernolactone significantly increased the activities of caspase 3 and caspase 7 in NTERA-2 cells at the concentrations of 2 *μ*g/mL and 4 *μ*g/mL (*P* < 0.0001) while paclitaxel, as the positive control, significantly increased the caspase 3 and caspase 7 activity at concentrations 0.25 *μ*g/mL, 0.5 *μ*g/mL, and 1 *μ*g/mL in a dose-dependent manner when compared to the untreated control cells (Figure 5).

#### 3.3.3. Effects of Vernolactone on the Expression of Apoptosis- and Autophagy-Related Genes

A large number of genes are reported to govern both apoptosis and autophagy pathways. Apoptosis and autophagy share some similarities. Thus, both are self-degradation pathways activated under cellular or environmental stress conditions. Even though, autophagy is a stress-adaptive response that prolongs cell survival, if it is allowed to proceed to completion, like apoptosis which can also lead to cell death [40].

**Figure 6:**
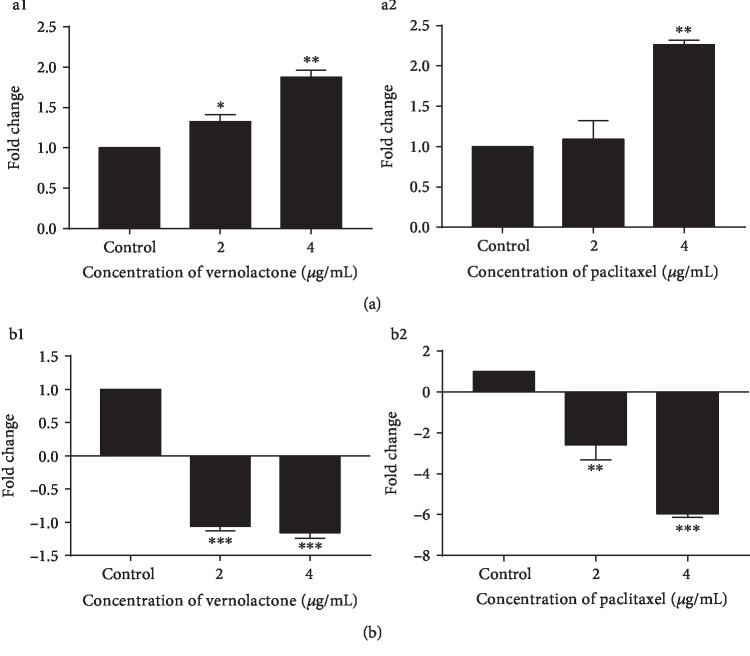
Expression of apoptosis-related genes in vernolactone- and paclitaxel-treated NTERA-2 cells. (a1, a2) Expression of *p53* in vernolactone- and paclitaxel-treated cells, respectively. (b1, b2) Expression of *Survivin* in vernolactone- and paclitaxel-treated cells, respectively. ^∗^*P* < 0.05, ^∗∗^*P* < 0.01, and ^∗∗∗^*P* < 0.001.

*p53* is a tumor suppressor and a transcription factor which transactivates genes that are essential for the induction of cell cycle arrest, DNA repair, cellular senescence, apoptosis, and autophagy [41, 42]. Further, *p53* can also downregulate the antiapoptotic gene *Survivin* at transcriptional and translational levels to induce apoptosis [43, 44]. Both vernolactone and paclitaxel indicated a significant (vernolactone—*P* < 0.05 and *P* < 0.01; paclitaxel—*P* < 0.01) upregulation at 2 and 4 *μ*g/mL doses at 24 h postincubation (Figure 6 a1 and a2) while a significant downregulation (vernolactone—*P* < 0.001 and *P* < 0.001, paclitaxel—*P* < 0.01 and *P* < 0.001) was observed in the expression of *Survivin* with the treatment of vernolactone and paclitaxel at both the doses tested. (Figure 6 b1 and b2).

**Figure 7:**
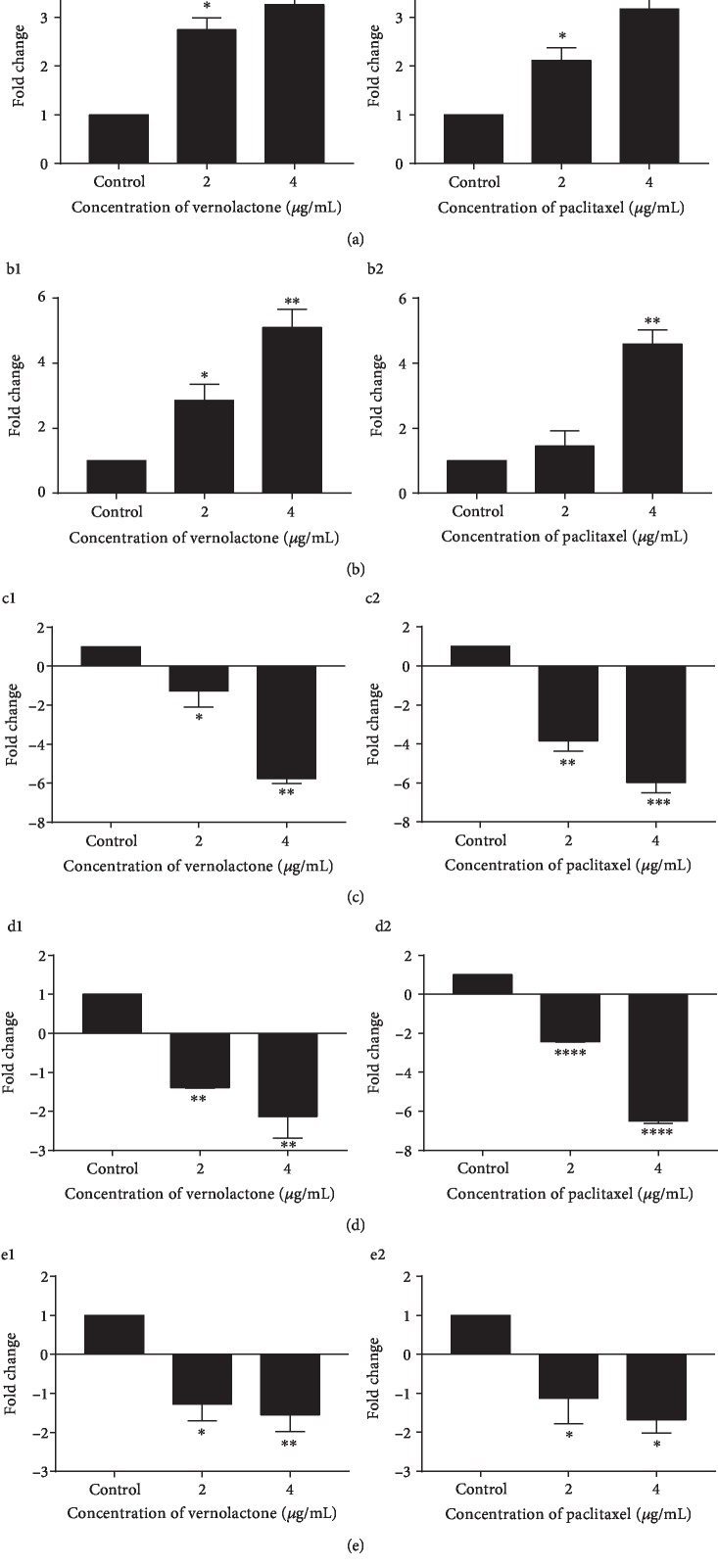
The expression of autophagy-related genes in vernolactone- and paclitaxel-treated NTERA-2 cells. (a1, a2) Expression of *LC3* after vernolactone and paclitaxel treatments, respectively. (b1, b2) Expression of *Beclin 1* after vernolactone and paclitaxel treatments, respectively. (c1, c2) Expression of *PI3K* after vernolactone and paclitaxel treatments, respectively. (d1, d2) Expression of *Akt* after vernolactone and paclitaxel treatments, respectively. (e1, e2) Expression of *mTOR* on vernolactone and paclitaxel treatments, respectively. ^∗^*P* < 0.05, ^∗∗^*P* < 0.01, ^∗∗∗^*P* < 0.001, and ^∗∗∗∗^*P* < 0.0001.

*LC3* (microtubule-associated protein 1A/1B-light chain 3) is the mammalian counterpart of autophagy gene ATG8 and is the most widely used marker in autophagosomes [45]. *Beclin 1*, the mammalian ortholog of yeast Atg6, is a tumor suppressor that plays a central role in autophagy and was recently found to be a BH3-only protein [46, 47]. The present study indicates that vernolactone, like paclitaxel, can mediate a significant (vernolactone—*P* < 0.05 and *P* < 0.01, paclitaxel—*P* < 0.05 and *P* < 0.01) dose-dependent upregulation of *LC3* and *Beclin 1* expression in NTERA-2 cells at 2 and 4 *μ*g/mL doses (Figure 7 a1, a2, b1, and b2). Activation of the PI3K/Akt/mTOR signaling pathway can inhibit autophagy since mTOR (mammalian target of rapamycin), which is the key negative regulator of autophagy [48]. The expression of *PI3K*, *Akt*, and *mTOR* was examined in the present study after treatment with vernolactone and paclitaxel which demonstrated a significant downregulation in their expression at the doses of 2 and 4 *μ*g/mL (Figure 7 c1, c2, d1, d2, e1, and e2).

Various studies have showed the role of the PI3K/Akt/mTOR pathway and its intermediates in the maintenance and survival of CSCs. Through the studies on prostate cancer, Chang et al. discovered that the PI3K/Akt/mTOR signaling pathway can activate CSC phenotypes and is associated with epithelial-mesenchymal transition (EMT) while Dubrovska et al. found out that the maintenance of prostate cancer stem-like cell was carried out via the PTEN/PI3K/Akt pathway [49, 50]. In breast cancer stem-like cells, *in vitro* colony formation ability and *in vivo* tumorigenicity are aided by the activation of this pathway [51]. Mutations or deletions of phosphatase and tensin homolog (*PTEN*), a negative regulator of the Akt pathway, are associated with several cancers including breast, brain, prostate, and leukemia that result in the resistance to conventional therapies [52].

According to the results obtained from the gene expression analysis of apoptosis- and autophagy-related genes, both vernolactone and paclitaxel can significantly induce apoptosis and autophagy in NTERA-2 cells via inhibiting the PI3K/Akt/mTOR pathway. This supports the fact that apoptosis and autophagy are possible mechanisms by which vernolactone mediates its anticancer effects.

### 3.4. Morphological Analysis of Induced Cellular Differentiation

**Figure 8:**
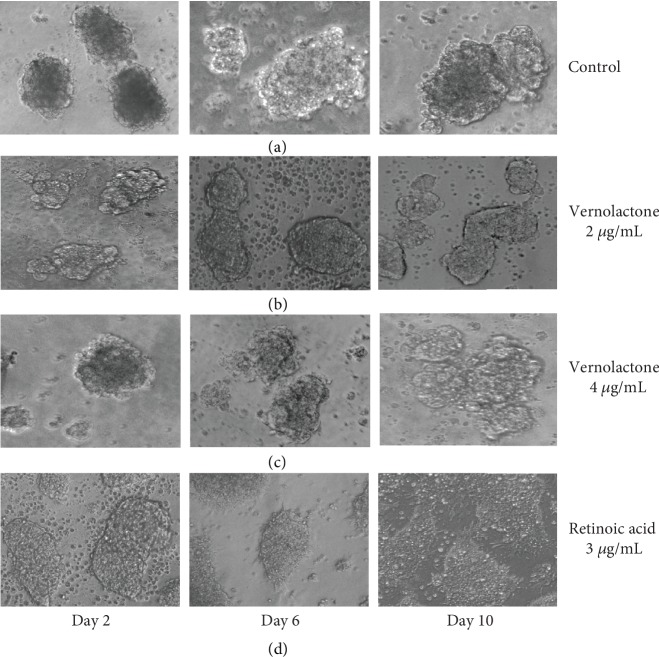
Phase contrast light microscopic images captured at different time intervals—morphological changes of (a) untreated control, (b) vernolactone (2 *μ*g/mL), (c) vernolactone (4 *μ*g/mL), and (d) retinoic acid (ATRA) (3 *μ*g/mL) (magnification 400x).

NTERA-2 is an extensively characterized cell line that can be committed to terminal differentiation into postmitotic neurons (NT2-N cells) in response to retinoic acid [53]. Differentiated NT2-N cells resemble human central nervous system (CNS) neurons, morphologically characterized by small rounded cell bodies with thin neuronal processes and expression of several neuronal markers [29, 54]. Since the previously established differentiation method (mono layer culture) by Pleasure et al. [54] is a time-consuming lengthy process, we followed the free floating sphere culture method described by Paquet-Durand et al. [28] which shortens the time required for differentiation. NTERA-2 cells proliferated as free floating clustered spheres in the medium. Upon treatment with ATRA, the clustered spheres showed thin processes projecting outside of the spheres over time. Considerable morphological changes were not observed in any of the vernolactone-treated NTERA-2-clustered spheres. Thus, these morphological analyses showed that vernolactone does not induce cellular differentiation in NTERA-2 cells (Figure 8).

## 4. Conclusion

The present study revealed that vernolactone isolated from *Vernonia zeylanica* can mediate its antiproliferative effects through apoptosis and autophagy in cancer stem-like cells with less antiproliferative effects in the noncancerous peripheral blood mononuclear cells (PBMC). Results of this study provided a strong rational to develop vernolactone as a new drug candidate for the treatment of cancer stem cells.

## Figures and Tables

**Table 1:** IC_50_ values (*μ*g/mL) of vernolactone and paclitaxel in NTERA-2 and peripheral blood mononuclear cells.

Cell type	Vernolactone			Paclitaxel		
	24 h	48 h	72 h	24 h	48 h	72 h
NTERA-2 cells	6.188	5.772	0.4808	4.958	1.789	0.1949
Peripheral blood mononuclear cells	143.2	84	68.81	256	195.9	113.6

## Data Availability

The data (results) used to support the findings of this study are included within the article.
